# Intersectional strategy to study cortical inhibitory parvalbumin-expressing interneurons

**DOI:** 10.1038/s41598-024-52901-y

**Published:** 2024-02-03

**Authors:** Rebeka Palicz, Bettina Pater, Pavel Truschow, Mirko Witte, Jochen F. Staiger

**Affiliations:** Center Anatomy, Institute for Neuroanatomy, University of Göttingen, Göttingen, Germany

**Keywords:** Neuroscience, Anatomy

## Abstract

Parvalbumin-expressing (PV) interneurons are key neuronal elements to a global excitatory-inhibitory balance in normal cortical functioning. To better understand the circuit functions of PV interneurons, reliable animal models are needed. This study investigated the sensitivity and specificity of the most frequently used *PV-Cre/tdTomato* mouse line in this regard. The colocalization of the transgene (tdTomato) with the parvalbumin protein, with *GAD1* (a conclusive inhibitory cell marker) and *Vglut1* (a conclusive excitatory cell marker) as well as with a marker for perineuronal nets (WFA) was assessed and a substantial proportion of layer 5 PV neurons was found to be excitatory and not inhibitory in the *PV-Cre/tdTomato* mouse. The intersectional transgenic mouse line *Vgat-Cre/PV-Flp/tdTomato* provided a solution, since no colocalization of tdTomato with the *Vglut1* probe was found there. In conclusion, the *Vgat-Cre/PV-Flp/tdTomato* mouse line seems to be a more reliable animal model for functional studies of GABAergic PV interneurons.

## Introduction

Cortical GABAergic neurons are a very diverse class of cells that vary widely in their molecular, physiological and morphological feature space^1–3^. Before the advent of transgenic mouse lines, this led to a confusing state of the field^4^. However, due to *IRES-Cre-knock-in* transgenic mouse lines^5^, the situation profoundly improved over the past decade^6–8^. Nevertheless, the specificity of transgene expression in newly generated mouse lines is often examined in a superficial manner. In order to validate these experimental model systems, a thorough characterization of the specificity and sensitivity of the transgene expression is warranted since time and again surprising observations have been reported^9–11^. Previously, we have provided a profound characterization of a VIP-expressing^12^ and a somatostatin-expressing mouse line^13^. Here, we set out to perform this characterization for two mouse models generated with the aim to genetically access the subpopulation of parvalbumin-expressing (PV) neurons, namely the *PV-Cre* and *Vgat-Cre/PV-Flp* mouse lines, which we crossed with the *Ai9* and *Ai65* reporter mouse lines, respectively^14^.

GABAergic interneurons can be classified into several subpopulations according to their protein expression, electrophysiological properties, morphology and synaptic patterns. Parvalbumin-expressing interneurons are fast-spiking GABAergic neurons, forming inhibitory synapses most frequently on the perisomatic region of their target cells^8,15–18^.

However, a largely ignored study has provided early evidence that parvalbumin can also be expressed by pyramidal cells in primary somatosensory cortex^19^. Therefore, experiments investigating circuitry and function of PV inhibitory interneurons^20–22^ need reliable animal models, since applied viral vectors for neuron manipulation or imaging would not distinguish between GABAergic and non-GABAergic neurons. With the recent advances in the generation of driver mouse lines using different specific recombinases, together with the appropriate intersectional reporter mouse lines, this confounder can now be remedied by combining a GABAergic neuron-specific driver line (*Vgat-Cre*)^5^ with a parvalbumin-specific driver line (*PV-Flp*) crossed with the *Cre*- and *Flp*-dependent reporter mouse line *Ai65*^14^.

Perineuronal nets (PNNs) are a specialized part of the extracellular matrix and surround different cell types, in the cortex of rodents mainly parvalbumin-expressing GABAergic interneurons^23^. Their appearance has been linked to the maturation of GABAergic inhibition during postnatal development^24–26^. They reduce the synaptic plasticity of parvalbumin cells by stabilizing synaptic contacts on these interneurons^27^. PNNs play in important role in memory consolidation^28–30^ and their disturbed formation contributes to several neurologic alterations like increased response to chronic pain or excitatory/inhibitory imbalance^31,32^, schizophrenia^33–35^, bipolar disorder^36^ and Alzheimer`s disease^37^. Here, we asked whether staining of PNNs will offer further insight into the characterization of the cell types labeled in the two transgenic mouse lines under study.

Therefore, in this study, we compared (i) *PV-Cre/tdTomato* and (ii) *Vgat-Cre/PV-Flp/tdTomato* mice in terms of transgenic red-fluorescent protein expression in parvalbumin-expressing interneurons by investigating the colocalization of tdTomato with parvalbumin, a pan-excitatory *(Vglut1)* and a pan-inhibitory *(Gad1)* marker as well as the wrapping of parvalbumin-expressing cells by perineuronal nets.

## Results

### Characterization of cortical parvalbumin-expressing neurons in the PV-Cre mouse line

At first, we mapped for 47 brain sections (in 8 mice) the average layer-specific distribution of tdTomato expressing cells. To present the results, we used descriptive statistics (mean, standard deviation and median, 25% percentile, 75% percentile, minimum and maximum to describe the distribution). In the *PV-Cre* mouse, the highest number of tdTomato-expressing cells was found in layer 4 (2857.6 ± 279.6 cells/mm^3^ barrel cortex), corresponding to a proportion of 28.7 ± 2.1% of all tdTomato-expressing cells. The lowest number of tdTomato-expressing cells were observed in layer 6 (1522.8 ± 349.4 cells/mm^3^ barrel cortex) and layer 5a (1499.8 ± 220.8 cells/mm^3^) corresponding to a proportion of 15.1 ± 2.8% and 15.1 ± 2.2% resp. of all tdTomato-expressing cells. Layer 5b contained 2468.8 ± 403.7 cells/mm^3^ corresponding to 24.7 ± 2.9%. Layer 2/3 contained 1617.3 ± 207.7 cells/mm^3^ barrel cortex. (Supplementary Fig. 1).

**Figure 1:**
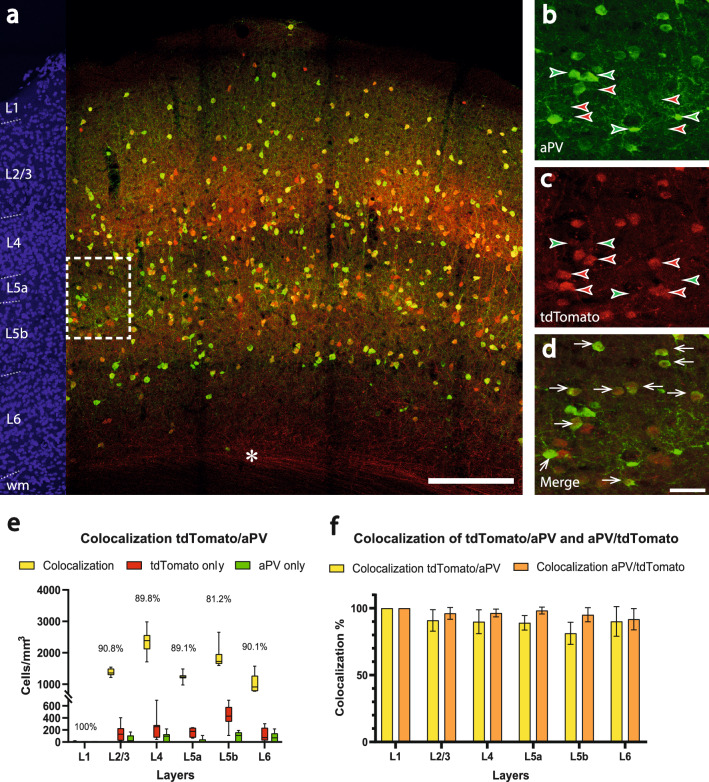
Colocalization of parvalbumin and tdTomato in the barrel cortex of the *PV-Cre/tdTomato* mouse. (**a**) Overview image showing immunoamplified transgenic tdTomato and immunohistochemical staining for parvalbumin. Red corresponds to tdTomato-signal, green corresponds to parvalbumin antibody labeling. On the left margin, cell nuclei are shown in blue (DAPI) for laminar delineation; wm: white matter. The asterisk indicates axons of tdTomato cells (putative parvalbumin-expressing pyramidal cells) leaving the cortex. Scalebar: 200 µm. (**b–d**) higher magnification of the boxed area in (**a**). (**b**) reveals the expression of parvalbumin (aPV, green), (**c**) shows the tdTomato signal (red) and (**d**) displays the colocalization of tdTomato and parvalbumin. The white arrows indicate colocalization, whereas green arrowheads label the cells expressing only parvalbumin protein and red arrowheads label the cells expressing only the tdTomato transgene. Scalebar: 30 µm. (**e**) Counts of colocalized (yellow; colocalization rate on top) and non-colocalized (green and red) cells in a layer-specific manner. (**f**) Colocalization rate of tdTomato with parvalbumin (indicating specificity, yellow) versus the colocalization rate of the parvalbumin antibody with tdTomato (indicating sensitivity, orange).

We defined colocalization as the overlap of the red fluorescent label, AlexaFluor 594 (corresponding to the genetically encoded tdTomato signal) with the green fluorescent label, AlexaFluor 488 (corresponding to parvalbumin antibody labeling) and used it as an assay to probe the specificity of Cre expression for parvalbumin-expressing neurons in this mouse line (n = 7 brain sections from 4 mice) (Fig. 1a). At first, we studied how many of the red fluorescent cells also have a green labeling to probe how specific tdTomato fluorescence is for parvalbumin-expressing cells in this mouse line. In 1 mm^3^ barrel cortex, there were 7846.6 ± 816.0 cells/mm^3^ showed a colocalization (white arrows in Fig. 1d) of tdTomato and parvalbumin, which resulted in a mean colocalization of 87.6 ± 6.7%. Furthermore, 348.4 ± 279.2 cells showed only parvalbumin expression (green arrowheads in Fig. 1b,c) and 1097.0 ± 561.8 cells showed only tdTomato expression (red arrowheads in Fig. 1b,c). Layer I was not considered in the analysis due to low cell body numbers (being in agreement with a virtual absence of PV cells in layer 1)^38^. The layer-specific distribution of cells showing a colocalization of the red and green fluorescent labels had the highest proportion in layer 2/3 (mean: 90.8 ± 8.0%) and the lowest in layer 5b (mean: 81.2 ± 8.3%) (Fig. 1e). The highest number of colocalized cells was observed in layer 4 (mean: 2357.7 ± 394.2, median: 2390.1, 25% percentile: 2111.2, 75% percentile: 2560.1, min: 1711.5, max: 2983.3), the lowest in layer 6 (mean: 1014.6 ± 299.0, median: 910.1, 25% percentile: 779.9, 75% percentile: 1269.3, min: 775.2, max: 1572.6). The highest number of non-colocalized cells was observed in layer 5b (mean: 423.1 ± 187.3, median: 430.3, 25% percentile: 338.1, 75% percentile: 581.37, min: 107.7, max: 693.3). After examining the colocalization of tdTomato with parvalbumin, we took the reverse perspective and observed the colocalization of the green fluorescent label with the red signal in our target cells (orange columns in Fig. 1f). The mean colocalization of parvalbumin antibody labeling with tdTomato expression as a measure of sensitivity corresponded to 95.6 ± 3.7% in the overall cortex.

These results suggest that the mouse model is sensitive, but its specificity needs to be discussed further (see Discussion “Technical considerations”). Since previously some weakly PV-expressing excitatory neurons had been described^19^, we next examined the potential excitatory nature of tdTomato-expressing cells, while at the same time verifying their assumed inhibitory phenotype. Toward this aim, the colocalization of tdTomato with inhibitory and excitatory cell markers was observed. We used the *Gad1* probe (glutamic acid decarboxylase 67 kD, the key enzyme necessary for GABA synthesis) as a marker for inhibitory cells and the *Vglut1* probe (vesicular glutamate transporter 1, the synaptic vesicle transporter of glutamate) as a marker for excitatory cells.

**Figure 2:**
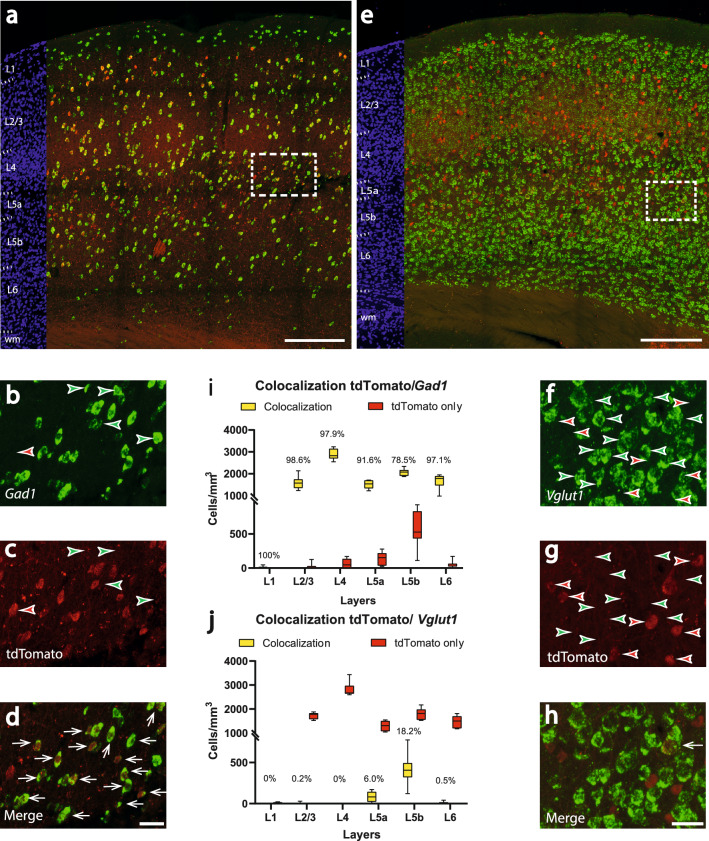
Colocalization of tdTomato with the inhibitory cell marker *Gad1* and the excitatory cell marker *Vglut1* within the barrel cortex of the *PV-Cre/tdTomato* mouse line. (**a**) Overview image showing transgene expression and in-situ hybridization for *Gad1*. Red corresponds to tdTomato signal, green corresponds to the *Gad1* probe. On the left margin, cell nuclei are shown in blue (DAPI) for laminar delineation; wm: white matter. Scalebar: 200 µm. (**b-d**) Higher magnification of the boxed area in **a**) revealing a high but not thorough colocalization (**d**) of *Gad1* (**b**) and tdTomato signal (**c**). Scalebar: 30 µm. (**e**) Overview image showing transgene expression and in-situ hybridization for *Vglut1*. Red corresponds to tdTomato-signal, green corresponds to the *Vglut1* probe. On the left margin, cell nuclei are shown in blue (DAPI) for laminar delineation; wm: white matter. Scalebar: 200 µm. (**f–h**) Higher magnification of the boxed area in (**e**) revealing a low but not absent colocalization (**h**) of *Vglut1* (**f**) and tdTomato signal (**g**). Scalebar: 30 µm. White arrows indicate colocalization, whereas green arrowheads label the cells expressing only the *Gad1* (**b** and **c**) or the *Vglut1* (**f**,**g**) probe. Please note, to keep Figs. 4f and 4g clear, not every cell showing the *Vglut1* probe was marked with a green arrowhead. Red arrowheads label the cells expressing only the tdTomato signal. (**i**) Cell counts of tdTomato/*Gad1*-colocalizing neurons show a peak expression of non-colocalized and thus presumably excitatory cells in layer 5b. (**j**) Complementary cell counts of tdTomato/*Vglut1*-colocalizing neurons show a peak expression of colocalized and thus excitatory cells in layer 5b. The colocalization rate is indicated on each yellow column.

For *Gad1* (n = 8 brain sections from 4 mice), most of the tdTomato labeled cells (red) showed colocalization with the *Gad1* probe (green) (Fig. 2a). In 1 mm^3^ barrel cortex, there were 839.3 ± 342.4 cells, which showed only tdTomato expression (red arrowheads in Fig. 2b,c). 9701.4 ± 807.2 cells/mm^3^ showed a colocalization of tdTomato and the *Gad1* probe (white arrows in Fig. 2d), which resulted in a mean colocalization of 92.1 ± 3.3%. The highest colocalization proportion was observed in layer 2/3 (mean: 98.5 ± 3.2%), the lowest in layer 5b (mean: 79.3 ± 8.0%) (Fig. 2i). The highest number of colocalized cells was seen in layer 4 (mean:2870.9 ± 246.7 cells/mm^3^, median: 2827.8, 25% percentile: 2702.5, 75% percentile: 3143.6, min: 2545.8, max: 3232.9), the lowest in layer 5a (mean: 1515.7 ± 189.2 cells/mm^3^, median: 1538.4, 25% percentile: 1333.4, 75% percentile: 1701.4, min: 1226.0, max: 1711.5). Layer 5b showed the highest number of tdTomato-only (non-colocalized) cells (mean: 565.4 ± 265.8 cells/mm^3^, median: 525.5, 25% percentile: 434.0, 75% percentile: 829.5, min: 110.3, max: 925.3).

For *Vglut1* (n = 8 brain sections from the same 4 mice as *Gad1* staining), only an exceedingly small amount of tdTomato cells (red) showed a colocalization with the *Vglut1* probe (green) (Fig. 2e). In 1 mm^3^ barrel cortex, there were 9142.0 ± 596.1 cells, which showed only tdTomato expression (red arrowheads in Fig. 2f,g). 508.9 ± 162.1 cells/mm^3^ showed a colocalization of tdTomato and the *Vglut1* probe (white arrow in Fig. 2h), which resulted in a mean colocalization of 5.3 ± 1.6%. The highest colocalization proportion was observed in layer 5b (mean: 18.2 ± 6.6%), the lowest in layer 4 (0%). The highest number of colocalized cells was seen in layer 5b (mean: 414.7 ± 185.3 cells/mm^3^, median: 406.2, 25% percentile: 319.2, 75% percentile: 492.9, min: 122.1, max: 772.7) (Fig. 2j). Thus, colocalized cells were mainly observed in layers 5a and 5b, exactly where we found the least colocalization proportion with *Gad1.*

These results predict that in the *PV-Cre* mouse line, any transgene expression will not be restricted to GABAergic interneurons but will also involve excitatory neurons mainly in layer 5b but also in layer 5a.

### Characterization of cortical parvalbumin-expressing neurons in the Vgat-Cre/PV-Flp mouse line

As a possible solution for obtaining a tool to exclusively study GABAergic PV cells, we investigated the colocalization profile of tdTomato-expressing cells in the *Vgat-Cre/PV-Flp* mouse using altogether 4 mice. In this mouse line, the transgene should be expressed only in GABAergic cells.

In the *Vgat-Cre* mouse, we tested the layer-specific distribution of tdTomato-expressing cells on 59 brain sections. The highest number of tdTomato-expressing cells was found in layer 4 (2532.9 ± 645.9 cells/mm^3^), which corresponds to a proportion of 29.7 ± 7.0% of all tdTomato-expressing cells. The lowest number of tdTomato-expressing cells was observed in layer 5a (1267.8 ± 351.1 cells/mm^3^), corresponding to a proportion of 14.8 ± 3.7%. Layer 5b contained 1822.2 ± 517.7 cells/mm^3^, corresponding to 21.2 ± 5.3%. Layers 2/3 and 6 contained 1365.1 ± 505.7 cells/mm^3^ and 1582.9 ± 465.3 cells/mm^3^ resp. (Supplementary Fig. 2).

**Figure 3:**
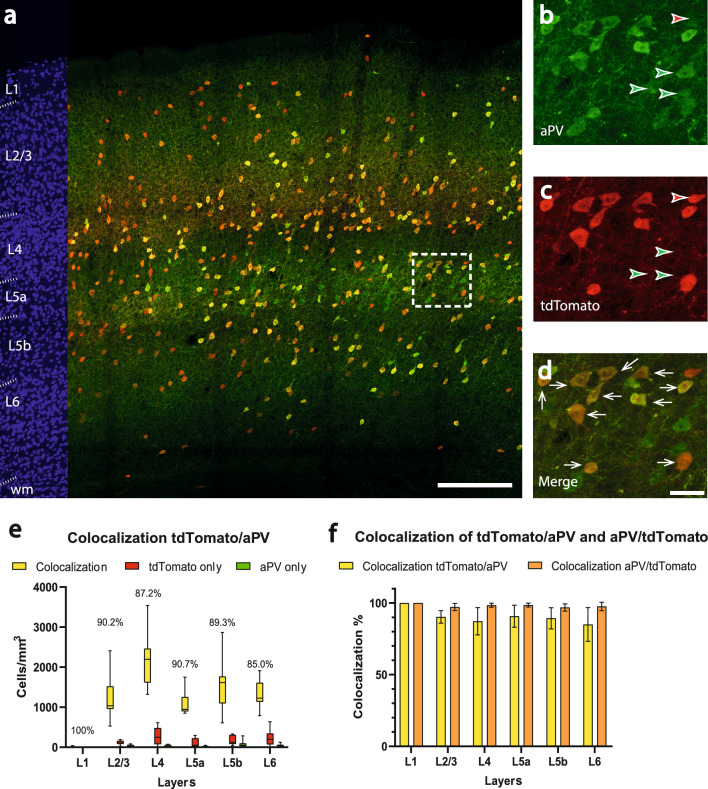
Colocalization of parvalbumin and tdTomato in the barrel cortex of the *Vgat-Cre*/*PV-Flp/tdTomato* mouse. (**a**) Overview image showing immunoamplified transgenic tdTomato and immunohistochemical staining for parvalbumin. Red corresponds to tdTomato signal, green corresponds to parvalbumin antibody labeling. On the left margin, cell nuclei are shown in blue (DAPI) for laminar delineation; wm: white matter. Scalebar: 200 µm. (**b–d**) higher magnification of the boxed area in (**a**). (**b**) reveals the expression of parvalbumin (green; aPV), (c) shows the tdTomato signal (red) and (**d**) displays the colocalization of tdTomato and parvalbumin. Scalebar: 30 µm. The white arrows indicate colocalization whereas green arrowheads label the cells expressing only parvalbumin protein and red arrowheads label the cells expressing only the tdTomato transgene. (**e**) Counts of colocalized (yellow; colocalization rate on top) and non-colocalized (green and red) cells in a layer-specific manner, applying PV immunohistochemistry. (**f**) Colocalization rate of tdTomato with the parvalbumin antibody (indicating specificity, yellow) versus the colocalization rate of the parvalbumin antibody with tdTomato (indicating sensitivity, orange).

For studying the colocalization of tdTomato with parvalbumin protein (Fig. 3a), we used 11 brain sections. In 1 mm^3^ barrel cortex, there were 193.2 ± 102.8 cells, which only showed parvalbumin expression (green arrowheads in Fig. 3b,c) and 924.2 ± 455.8 cells that showed only tdTomato expression (red arrowheads in Fig. 3b,c). 7116.5.6 ± 1532.5 cells/mm^3^ showed a colocalization of tdTomato and parvalbumin (white arrows Fig. 3d), which resulted in a mean colocalization of 87.9 ± 6.2%. The highest colocalization proportion was observed in layer 5a (mean: 90.7 ± 7.7%), the lowest in layer 6 (mean: 85.0 ± 11.8%). The highest number of colocalized cells was seen in layer 4 (mean: 2224.4 ± 622.2 cells/mm^3^, median: 2194.0, 25% percentile: 1613.8, 75% percentile: 2463.5, min: 1322.7, max: 3539.8), the lowest in layer 5a (mean: 1129.4 ± 321.3 cells/mm^3^, median: 944.8, 25% percentile: 893.9, 75% percentile: 1253.5, min: 853.2, max: 1752.9) (Fig. 3e). The layer-specific analysis on the colocalization showed no significant difference with the *PV-Cre* mouse line (overall: p = 0.9298, for layer2/3: p = 0.6590, for layer 4: p = 0.7242, for layer 5a: p = 0.8397, for layer 5b: p = 0.0853, for layer 6: p = 0.3394), indicating that the proportional distribution of the neurons across laminar compartments is very similar. Also here, we took the reverse perspective and observed the colocalization of the green fluorescent label with the red signal in our target cells as a measure of sensitivity (orange columns in Fig. 3f). The mean colocalization of parvalbumin antibody labeling with tdTomato expression corresponded to 97.3 ± 1.4% in the overall cortex.

**Figure 4:**
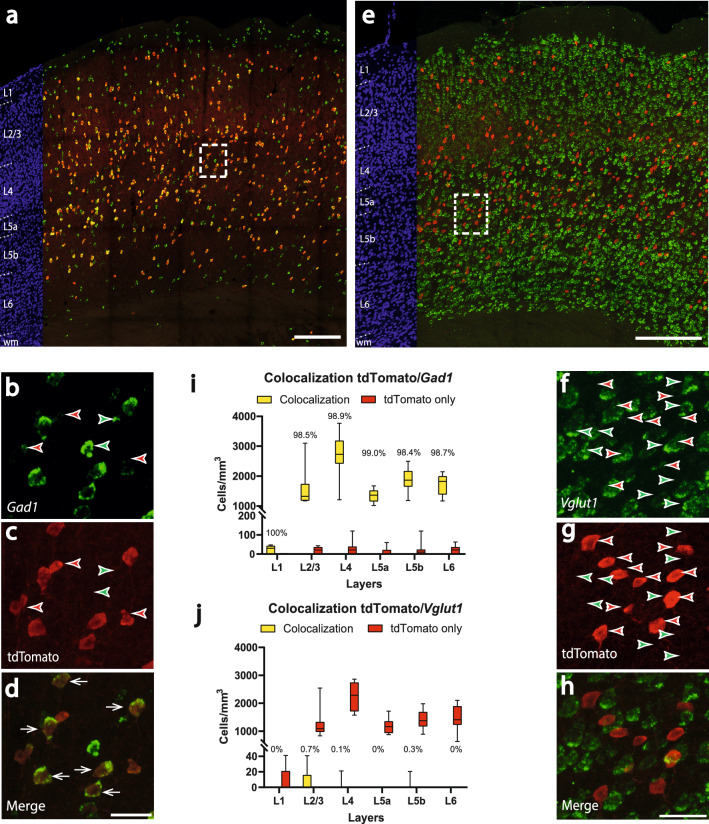
Colocalization of tdTomato with the inhibitory cell marker *Gad1* and the excitatory cell marker *Vglut1* within the barrel cortex of the *Vgat-Cre/PV-Flp/tdTomato* mouse line. **a**) Overview image showing transgene expression and in-situ hybridization for *Gad1*. Red corresponds to tdTomato signal, green corresponds to the *Gad1* probe. On the left margin, cell nuclei are shown in blue (DAPI) for laminar delineation; wm: white matter. Scalebar: 200 µm. **b-d)** Higher magnification of the boxed area in **a**) revealing a high but not thorough colocalization (**d**) of *Gad1* (**b**) and tdTomato signal (**c**). Scalebar: 30 µm. **e)** Overview image showing transgene expression and in-situ hybridization for *Vglut1*. Red corresponds to tdTomato signal, green corresponds to the *Vglut1* probe. On the left margin, cell nuclei are shown in blue (DAPI) for laminar delineation; wm: white matter. Scalebar: 200 µm. **f–h)** Higher magnification of the boxed area in **e**) revealing a virtually non-existent colocalization rate (**h**) of *Vglut1* (**f**) and tdTomato signal (**g**). Scalebar: 30 µm. White arrows indicate colocalization, whereas green arrowheads label the cells expressing only the *Gad1* (**b** and **c**) or the *Vglut1* (**f** and **g**) probe. Red arrowheads label the cells expressing only the tdTomato signal. **i)** Cell counts of tdTomato/*Gad1*-colocalizing neurons show a nearly complete overlap of the two signals. **j)** Complementary cell counts of tdTomato/*Vglut1*-colocalizing neurons hardly show any overlap. The colocalization rate is indicated on each yellow column.

Concerning the inhibitory or excitatory nature of the tdTomato-cells, the same analysis was implemented as for the *PV-Cre* mouse. Also here, most cells showed a colocalization of tdTomato and *Gad1* (n = 8 brain sections) (Fig. 4a). In 1 mm^3^ barrel cortex, there were 99.5 ± 120.1 cells, which showed only tdTomato expression (red arrowheads in Fig. 4b,c). The mean number of tdTomato cells colocalizing with *Gad1* was 9360,8 ± 1038,6 cells/mm^3^, (white arrows in Fig. 4d), which corresponds to a mean colocalization of 98.9 ± 1.4%. This is a significantly higher proportion than in the *PV-Cre* mouse, which was 92.1 ± 1.4% (p = 0.0003). The highest colocalization proportion was observed in layer 5a (mean: 99.0 ± 1.9%), the lowest in layer 5b (mean: 98.4 ± 3.1%). The highest number of colocalized cells was seen in layer 4 (mean: 2726.8 ± 751.0 cells/mm^3^, median: 2728.5, 25% percentile: 2417.1, 75% percentile: 3183.31, min: 1209.9, max: 3764.0), the lowest in layer 5a (mean: 1342.4 ± 215.7 cells/mm^3^, median: 1361.2, 25% percentile: 1154.74, 75% percentile: 1521.2, min: 121.2, max: 1671.5) (Fig. 4i). In the layer-specific distribution, layers 5a and 5b showed a significant higher colocalization proportion, than in the *PV-Cre* mouse line (91.6 ± 5.7% vs. 99.0 ± 1.9% p = 0.0011 for L5a and 81.2 ± 8.3% vs. 98.4 ± 3.1% p = 0.0003 for L5b).

For the excitatory cell marker *Vglut1* (n = 8 brain sections), colocalization was virtually non-existent (Fig. 4e,h). In 1 mm^3^ barrel cortex, only 12.9 ± 24.3 cells were colocalized and 7523.4 ± 1245.2 cells showed a tdTomato expression (red arrowheads in Fig. 4f,g). This corresponds to a mean colocalization of 0.2 ± 0.4% (Fig. 4j), which we consider as biological noise. The colocalization for tdTomato and *Vglut1* was significantly lower in the *Vgat-Cre/PV-Flp* mouse, than in the *PV-Cre* mouse line (overall: 5.3 ± 1.6% vs. 0.2 ± 0.4% p = 0.0002, for L5a 6.1 ± 4.7% vs. 0% p = 0.0014, for L5b 18.2 ± 6.6% vs. 0.3 ± 0.8% p = 0.0002).

These results imply that the *Vgat-Cre/PV-Flp* mouse line is highly specific for targeting transgene expression to GABAergic parvalbumin-expressing interneurons.

### Perineuronal nets

For this part of the study, we wanted to leverage the known tight association of perineuronal nets with PV neurons^39–43^ to inquire whether they have a potential to distinguish between inhibitory and excitatory PV neurons. Thus, we compared the PNN-enwrapping of tdTomato-cells in both mouse lines. For this purpose, we used WFA (Wisteria floribunda agglutinin), a lectin labeling chondroitin sulfate proteoglycans as a major component of the PNN^25,44,45^.

**Figure 5:**
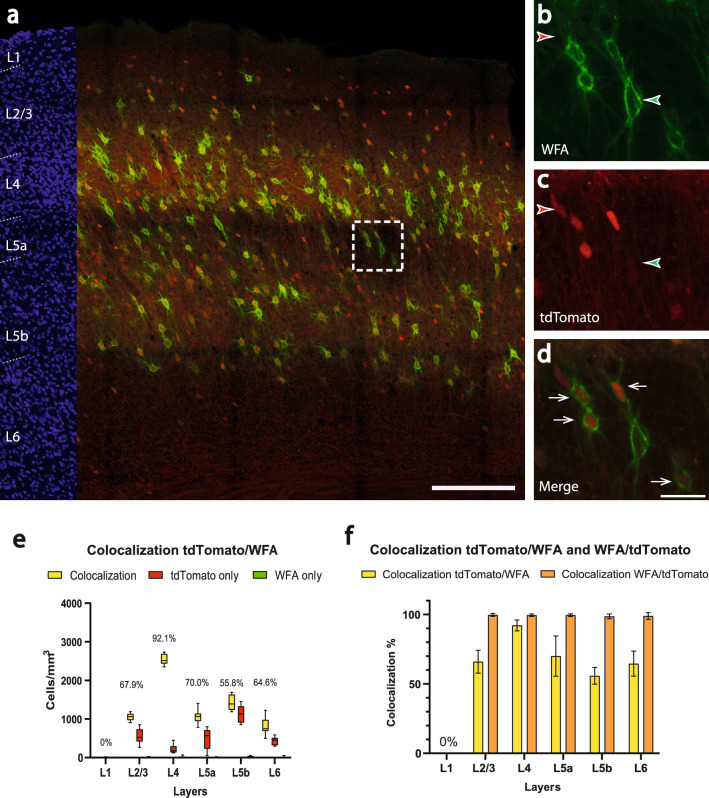
Colocalization of perineuronal nets and tdTomato in the barrel cortex of the *PV-Cre/tdTomato* mouse. (**a**) Overview image showing immunoamplified transgenic tdTomato and histochemical staining for WFA. Red corresponds to tdTomato signal, green corresponds to WFA labeling. On the left margin, cell nuclei are shown in blue (DAPI) for laminar delineation; wm: white matter. Scalebar: 200 µm. **b-d)** higher magnification of the boxed area in **a**). **b)** reveals the expression of WFA (green), **c)** shows the tdTomato signal and **d)** displays the colocalization of tdTomato and WFA. Scalebar: 30 µm. The white arrows indicate colocalization, whereas green arrowheads label the WFA-surrounding and red arrowheads label the cells expressing only the tdTomato transgene. **e)** Counts of colocalized (yellow; colocalization rate on top) and non-colocalized (green and red) cells in a layer-specific manner. **f)** Colocalization rate of tdTomato with WFA (yellow; indicating that not every PV cell possesses a perineuronal net) versus the colocalization rate of WFA with tdTomato (orange; indicating that all perineuronal nets were around PV cells).

First, PNN were studied in the *PV-Cre/tdTomato* mouse (n = 8 brain sections). The red fluorescent tdTomato-cells were surrounded by the green fluorescent WFA. This configuration corresponds to a spatial codistribution, but in order to have a uniform presentation of our findings, we keep the term colocalization for our figures and result descriptions. Remarkably, the green signal of WFA could be seen nearly exclusively around red fluorescent tdTomato-cells (Fig. 5a). In the considered brain sections, there were 2839.0 ± 603.4 cells/mm^3^, which showed only tdTomato expression, without WFA-surrounding (red arrowheads in Fig. 5b,c). A mean number of cells amounting to 6909.6 ± 587.3 cells/mm^3^ were colocalized (white arrows in Fig. 5d). This corresponds to a mean colocalization of 70.9 ± 5.6%. The highest colocalization proportion was observed in layer 4 (mean: 92.1 ± 3.9%), the lowest in layer 5b (mean: 55.8 ± 6.0%). The highest number of colocalized cells was seen in layer 4 (mean: 2538.0 ± 136.1 cells/mm^3^, median: 2515.3, 25% percentile: 2434.6, 75% percentile: 2687.5, min: 2351.1, max: 2733.4), the lowest in layer 6 (mean: 808.8 ± 224.9 cells/mm^3^, median: 748.4, 25% percentile: 692.5, 75% percentile: 979.3, min: 500.7, max: 1225.9) (Fig. 5e). From the reverse perspective, the mean colocalization of WFA with tdTomato corresponds to 99.2 ± 0.9% for the overall cortex (orange columns in Fig. 5f).

**Figure 6:**
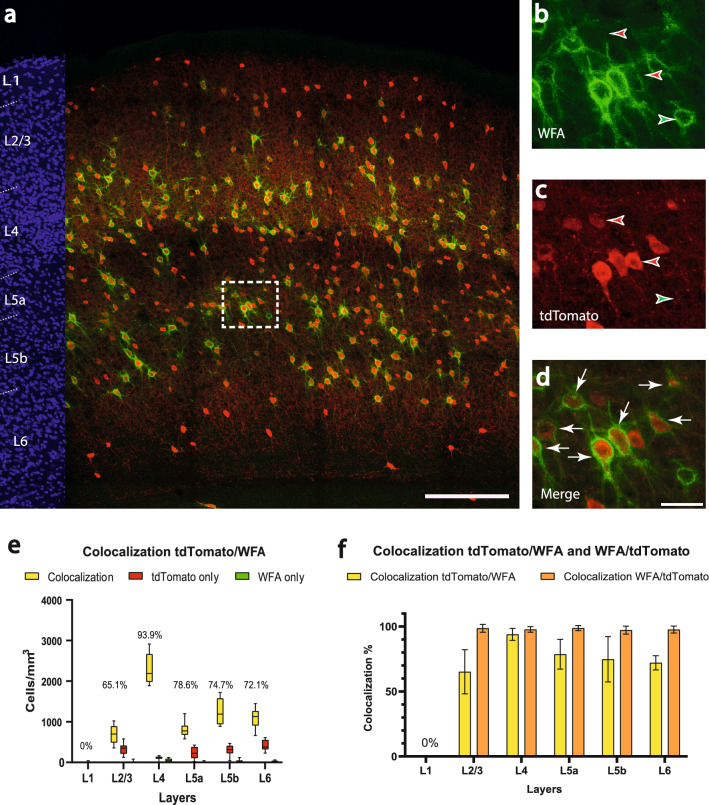
Colocalization of perineuronal nets and tdTomato in the barrel cortex of the *Vgat-Cre/PV-Flp/tdTomato* mouse. **a**) Overview image of immunoamplified transgenic tdTomato and histochemical staining for WFA. Red corresponds to tdTomato signal, green corresponds to WFA labeling. On the left margin, cell nuclei are shown in blue (DAPI) for laminar delineation; wm: white matter. Scalebar: 200 µm. (**b-d**) higher magnification of the boxed area in (**a**). **b)** reveals the expression of WFA (green), **c)** shows the tdTomato signal and **d)** displays the colocalization of tdTomato and WFA. Scalebar: 30 µm. The white arrows indicate colocalization, whereas green arrowheads label the WFA-surrounding and red arrowheads label the cells expressing only the tdTomato transgene. **e)** Counts of colocalized (yellow; colocalization rate on top) and non-colocalized (green and red) cells in a layer-specific manner. (**f**) Colocalization rate of tdTomato with WFA (yellow; indicating that not every PV cell possesses a perineuronal net) versus the colocalization rate of WFA with tdTomato (orange; indicating that all perineuronal nets were around PV cells).

The same analysis on PNN was performed in the *Vgat-Cre/PV-Flp* mouse (n = 8 brain sections) (Fig. 6a). We found 1421.4 ± 384.1 cells showing only tdTomato expression in 1 mm^3^ barrel cortex (red arrowheads in Fig. 6b,c). There were 5984.0 ± 722.1 colocalized cells in 1 mm^3^ barrel cortex (white arrows in Fig. 6d), corresponding to a mean colocalization of 80.7 ± 5.5%. This was significantly higher than in the *PV-Cre* mouse line (70.9 ± 5.6% vs. 80.7 ± 5.5% p = 0.003). The highest colocailzation proportion was observed in layer 4 (mean: 93.9 ± 4.6%), the lowest in layer 2/3 (mean: 65.1 ± 16.9%). The highest number of colocalized cells was seen in layer 4 (mean: 2308.7 ± 370.9 cells/mm^3^, median: 2190.9, 25% percentile: 1983.9, 75% percentile: 2667.1, min: 1889.3, max: 2916.6), the least colocalizations were found in layer 2/3 (mean: 694.2 ± 230.0 cells/mm^3^, median: 697.8, 25% percentile: 488.6, 75% percentile: 892.1, min: 359.2, max: 1022.4) (Fig. 6e). Layer 5b showed a significantly higher colocalization than in the *PV-Cre* mouse line (55.8 ± 6.0% vs. 74.7 ± 17.4% p = 0,0104). From the reverse perspective, the mean colocalization of WFA with tdTomato corresponds to 97.8 ± 1.2% for the overall cortex (orange columns in Fig. 6f).

These results are in line with those of the first part of the study. In the *PV-Cre* mouse, layer 5b has the lowest colocalization proportion (55.6 ± 6.0%) and the highest number of tdTomato-only (non-colocalized) cells (1137.1 ± 21.5 cells/mm^3^). Considering that PNN surround almost exclusively parvalbumin-expressing GABAergic interneurons in the barrel cortex (99.2 ± 0.9% and 97.8 ± 1.2% in *PV-Cre* and *Vgat-Cre/PV-Flp,* respectively), we can assume, that in the *PV-Cre* mouse line, excitatory cells coexpressing parvalbumin are present, especially in layer 5b. This subgroup of cells disappears in the *Vgat-Cre/PV-Flp* mouse, since here layer 2/3 has the lowest colocalization proportion (65.1 ± 16.9%).

## Discussion

In contrast to many previous BAC transgenic mice^46,47^, *IRES-Cre knock-in* mice like *PV-Cre* are generally considered to have a highly specific transgene expression with respect to the targeted cell population^5,48^. However, once in a while, at least for some brain areas, off-target expression^10^ or low sensitivity^11^ has been reported. This points to the prevailing need to characterize each transgenic mouse line for specificity and sensitivity of its transgene expression, in order to be able to draw firm conclusions from functional manipulation experiments.

Parvalbumin (PV)-expressing GABAergic interneurons have a crucial role in maintaining the excitation-inhibition balance in the cortex and contribute to the development of psychiatric conditions like autism, schizophrenia or Alzheimer`s disease^49–52^. Therefore, it is very important to find reliable animal models specific and sensitive for this cell type. In the present study, we compared the widely used *PV-Cre/tdTomato* and the more recently introduced *Vgat-Cre/PV-Flp/tdTomato* mouse lines with regard to specificity of genetically targeted recombinase expression for PV-expressing interneurons. We show that only with an intersectional approach^14^ the physiologically occurring population of PV-expressing pyramidal cells in L5^3,19^ can be excluded from functional manipulations like optogenetics or chemogenetics, in order to achieve an unbiased readout.

### Technical considerations

Ideally, one would like to have a highly sensitive and specific transgenic animal model, which labels 100% of all target cells as verified by protein or mRNA detection of a marker molecule that characterizes the neurons of interest. However, due to inherent technical limitations caused by histological processing, neither immunohistochemistry nor in situ* hybridization* (*ISH*) does achieve this performance. Antibodies can suffer from fixation masking epitopes or from poor penetration^53,54^. In the same direction, RNA probes are sensitive to a number of experimental factors that might compromise the readout^55^. As we also used lectin histochemistry, here the sugar moieties that are detected by a single lectin may not be inclusive for all molecular compositions of perineuronal nets that do exist^23^.

What other reasons could explain the roughly 12–13% of “unidentified” tdTomato cells in both mouse lines? One possibility could be that they underwent Cre-mediated recombination during early development but later in life lost their ability to express parvalbumin due to unknown molecular biological mechanisms. A similar phenomenon was suggested by Hu et al.^9^ for the *SOM-Cre* mouse line.

Another explanation could be the activity-dependent expression of the parvalbumin protein. Donato et al. have observed two populations of parvalbumin-expressing cortical interneurons: cells with a low parvalbumin-expression and a low excitatory-inhibitory synaptic density ratio and cells with a high parvalbumin-expression and a high excitatory-inhibitory synaptic density ratio^56^. According to this phenomenon, cells expressing the tdTomato signal which are negative for the parvalbumin antibody may be parvalbumin-expressing cells having a low protein expression due to low activation and therefore escape detection by the antibody labeling. This could also explain, why we still have ~ 12–13% of tdTomato + /aPV- cells in the Vgat-Cre/PV-Flp mouse line where the parvalbumin-expressing excitatory cells are eliminated. Furthermore, Filice et al. have shown, that in ASD mouse models, the number of parvalbumin interneurons is unchanged, while parvalbumin protein and PValb mRNA levels decrease, proving the existence of parvalbumin-expressing cells with a very low protein expression level^57^.

We also want to state here that in years of studying tdTomato-labeled cells in the *PV-Cre*/tdTomato mouse line with patch clamp recordings and subsequent single cell reconstruction, all neurons in all layers were fast spiking and showed a basket-cell like morphology^58^ (Preuss et al., unpublished observations).

Keeping this in mind, we will now carefully consider the results of our colocalization studies.

### Colocalization of the tdTomato signal with the parvalbumin antibody, glutamic acid decarboxylase 67 (Gad1) probe and vesicular glutamate transporter 1 (Vglut1) probe

In the *PV-Cre/tdTomato* mouse, the mean colocalization ratio of tdTomato with parvalbumin corresponded to 87.6 ± 6.7%. The mean colocalization of parvalbumin with tdTomato to 95.6 ± 3.7%. Assuming that ~ 12% of tdTomato-labeled neurons that did not express parvalbumin are a consequence of too low sensitivity of the antibody, we tentatively conclude that this mouse line is sensitive and specific for cortical PV neurons. To further investigate the inhibitory or potential excitatory nature of the tdTomato-expressing cells, their colocalization with *Gad1* and *Vglut1*, respectively, was studied. The mean colocalization of tdTomato with *Gad1* corresponded to 92.1 ± 3.3%, the mean colocalization of tdTomato with *Vglut1* to 5.3 ± 1.6%, pointing to a small but reliable excitatory neuron population. Interestingly, the highest number of tdTomato/*Vglut1* colocalizing cells and the highest colocalization ratio was found in layer 5b (414.7 ± 1885.3 cells/mm^3^ and 18.2 ± 6.6% resp.), which is consistent with previous findings describing parvalbumin-expressing cells, that are negative for GABA^59^ and found to be pyramidal cells^19^. Our study now provides a quantification of the proportion of these parvalbumin-expressing excitatory cells (5.3 ± 1.6%) averaged across all layers and revealed their-specific distribution by showing, that most of these cells are in layers 5a (6.0 ± 4.7%) and 5b (18.2 ± 6.6%).

In *Vgat-Cre/PV-Flp/tdTomato* mice, the mean colocalization of tdTomato with parvalbumin corresponded to 87.8 ± 6.6% and the mean colocalization of parvalbumin with tdTomato to 97.6 ± 1.0%. These figures, under the same assumption as above, also lead to the conclusion that this mouse line is sensitive and specific for PV cells. But did it also rid us from the excitatory cell component?

The colocalization of tdTomato and *Vglut1* corresponded to 0.2 ± 0.4% (versus 98.9 ± 1.4% colocalization with *Gad1*), so we have good evidence that, in this intersectional mouse line, the pyramidal neurons coexpressing parvalbumin were excluded from tdTomato expression. This is due to the molecular biological features of the *Vgat-Cre/PV-Flp/tdTomato* mouse line, which equal a biological “and”-gate^60^.

Another matter of caveat is the fact, that we used predominantly male animals in this study. Retrospectively, we consider this as scientifically undesirable. However, studies have shown that for histology data there were no sex differences and that females were not more variable than males^61^. Other studies have shown that the variability of gene expression was similar in males and females^62^. The predominance of male animals in our study had therefore most probably not much, if any, impact on our results.

### Codistribution with WFA

Perineuronal nets (PNNs) are part of the extracellular matrix surrounding the cell bodies and proximal dendrites of certain neurons^23^. Thus, the term colocalization here means codistribution in the immediate vicinity if the target cell`s plasma membrane. In different regions of the central nervous system, the cell types surrounded by PNNs are different: in the neocortex, mainly the parvalbumin-expressing GABAergic interneurons are surrounded by the PNNs^43,63^. However, in certain regions of the hippocampus^64^, PNNs surround excitatory and inhibitory cells.

In the neocortex of the mouse, the parvalbumin-expressing neurons are the main cell type surrounded by perineuronal nets^23^. This is consistent with our findings, since the mean colocalization of WFA with tdTomato corresponded to 99.2 ± 0.9% in the *PV-Cre/tdTomato* mouse and to 97.8 ± 1.2% in the *Vgat-Cre/PV-Flp/tdTomato* mouse. The mean colocalization of tdTomato with WFA corresponded to 70.9 ± 5.6% in the *PV-Cre/tdTomato* mouse and to 80.7 ± 5.5% in the *Vgat-Cre/PV-Flp/tdTomato* mouse, which shows a significant difference (p = 0.003). Thus, the large majority of the PV-cells have a PNN-ensheathment. Those that do not could be chandelier cells, which also would explain the pronounced non-colocalization in layer 2/3, which is home to many of these cells^65^. Altogether, our visualization of PNNs did not disclose a means to distinguish between GABAergic and glutamatergic PV neurons.

However, since not every type of perineuronal net can be visualized with WFA^66^, future studies using different lectins or antibodies might reveal strategies that could substitute for the laborious intersectional breeding strategy.

Summing up the results, our study has shown that the *Vgat-Cre/PV-Flp/tdTomato* mouse line is a more reliable animal model for experiments investigating the synaptic connections and functions of parvalbumin-expressing GABAergic interneurons^6,8^, since it rules out layer 5 pyramidal cells coexpressing parvalbumin. Future studies using the *PV-Cre/tdTomato* mouse line have to consider L5 pyramidal cells as a putative confounder.

## Methods

All experiments were carried out according to the German guidelines of animal care. The experimental protocol was approved by the Niedersächsische Landesamt für Verbraucherschutz und Lebensmittelsicherheit (LAVES; 33.19-42502-04-15/1897). The animals were kept under a 12 h day/12 h night cycle and had access to food and water ad libitum.

All experiments and methods were carried out in accordance with ARRIVE guidelines.

Eight adult *PV-Cre/tdTomato* mice (age: 4–8 months, weight: 20–30 g, 7 males, 1 female; 4 of these animals were used for immunohistochemical staining, another 4 for fluorescent in situ hybridization) and four *Vgat-Cre/PV-Flp/tdTomato* mice (age: 4 months, weight: 20–30 g, all males; the same animals were used for immunohistochemical staining and in situ hybridization) were used for this study. The use of a control group or randomization was not applicable in this study. *PV-Cre/tdTomato* mice were generated by crossing homozygous *Pvalb-IRES-Cre* (B6;129P2-Pvalbtm1(cre)Arbr/J, The Jackson Laboratory, Bar Harbor, USA) mice with homozygous *Ai9* mice (B6.Cg-Gt(ROSA)26Sortm9(CAG-tdTomato)Hze/J, The Jackson Laboratory). For generating *Vgat-Cre/PV-Flp/tdTomato* mice we first crossed homozygous *Vgat-IRES-Cre* ((Slc32a1tm2(cre)Lowl/J, The Jackson Laboratory) with homozygous *Pvalb-2A-FlpO-D* (B6.Cg-Pvalbtm4.1(FLPo)Hze/J, The Jackson Laboratory). Offspring heterozygous for *Vgat-Cre/PV-Flp* were further bred to obtain homozygous mice, which were crossed with *Ai65* mice (B6;129S-Gt(ROSA)26Sortm65.1(CAG-tdTomato)Hze/J, The Jackson Laboratory).

All mice were transcardially perfused with 4% paraformaldehyde (PFA) in 0.1 M phosphate buffer (PB, pH 7.4) after an intraperitoneal injection of ketamine (0.1 ml/10 g bodyweight). After the perfusion, the brains were removed from the skull and underwent postfixation (2 h in PFA at 4 °C). The brains used for immunohistochemistry were washed 2 × 15 min in 0.1 M PB pH 7.4 and stored at 4 °C until cutting. The brains for *fluorescent *in situ* hybridization* (*FISH)* were immersed in a cryoprotective solution (20% sucrose solution in 0.01 M PB with 0.9% NaCl, PBS, pH 7.4), stored overnight at 4 °C and were shockfrozen with isopentane at − 40 °C and stored at − 80 °C until cutting.

### Parvalbumin-immunohistochemistry and lectin histochemistry

The brains were cut in 40 µm-thick sections through the barrel cortex with a vibratome (VT 1200S, Leica, Wetzlar, Germany). The sections were washed 2 × 15 min in 0.05 M Tris buffer (TB, pH 7.6), 2 × 15 min 0.05 M Tris-buffered saline (TBS) pH 7.6 and 2 × 15 min with TBS and 0.5% Triton X-100 (TBST, pH 7.6) at room temperature. The sections were then blocked with 0.25% bovine serum albumin (BSA; Carl Roth, Karlsruhe, Germany) and 10% normal goat serum (NGS; Jackson Immuno-Research Laboratories, West Grove, Pennsylvania, USA) in TBST (0.05 M, pH 7.6). The primary antibody rabbit-anti-PV 25 (Swant, Martly, Switzerland) and the biotinylated lectin Wisteria floribunda agglutinin (WFA; Sigma-Aldrich, Munich, Germany) were diluted in the blocking solution to 1:5000 and 1:2000, respectively. Three brain sections were stained for each label, with the sections being incubated for 72 h at 4 °C. The sections were then washed 4 × 15 min with TBST (0.05 M, pH 7.6) at room temperature. After washing, the sections were incubated in the secondary antibody, goat-anti-rabbit Alexa 488 (Life Technologies, Darmstadt, Germany) diluted 1:500 in TBST (0.05 M, pH 7.6) or in Streptavidin Alexa Fluor 488 (Thermo Fisher Scientific, Massachusetts, USA) diluted 1:300 in TBST (0.05 M, pH 7.6) for 4 h at room temperature. The sections were washed after this incubation for 2 × 15 min in TBST (0.05 M, pH 7.6) and 1 × 15 min in TBS (0.05 M, pH 7.6) at room temperature. To display the cell nuclei, sections were also stained with 4′-5-diamidino-2-phenylindole (DAPI, Life Technologies, Darmstadt, Germany) diluted 1:5000 in TBS (0.05 M, pH 7.6) for 5 min at room temperature. After staining the nuclei, the sections were washed 1 × 15 min in TBS (0.05 M, pH 7.6) and 2 × 15 min in TB (0.05 M, pH 7.6) and stored in TB (0.05 M, pH 7.6) overnight at 4 °C. Then the sections were mounted on slides and covered with coverslips coated with Aqua Poly-Mount (Polyscience, Warrington, Pennsylvania, USA).

### Fluorescent-in-situ-hybridization (FISH)

Mouse brains were stored at − 80 °C and cut into 40 µm-thick sections through the barrel cortex (Bregma − 1.06 mm to − 1.94 mm) with a cryostat (Leica CM3050 S, Nuβloch, Germany) at − 18 °C. Four *PV-Cre/tdTomato* and 4 *Vgat-Cre/PV-Flp/tdTomato* mouse brain sections were cut, collected in PBS (pH 7.4) and put in a multiwell-plate after washing with PBS (0.1 M, pH 7.4). To increase the permeability of the tissue, the sections were treated with 1% H_2_O_2_ in methanol for 20 min, followed by quenching in 0.2 M HCl for 8 min and finally treated with proteinase K dissolved in Tris–HCl/EDTA (10 mg/mL) for 10 min. The sections were postfixed in 4% PFA in 0.1 m PB (pH 7.4) for 20 min at 0 °C. After 10 min of quenching in triethanolamine/HCl (0.1 M, pH 8), 2.4 µL/mL acetic anhydride was added. The sections were washed in PBS (0.1 M, pH 7.4) for 3 × 15 min and in Standard-Saline-Citrate buffer (2 × diluted SSC) (pH 7.1) for 1 × 15 min. The sections were incubated in SSC in hybridization buffer 1:1 (hybridization buffer: 50% deionized formamide, SSC buffer, 5% dextrane sulfate, 250 µg/mL Hss-DNA, 100 µg/mL, t-RNA and Denhardt`s solution) at room temperature. Incubation with the hybridization buffer followed for 60 min at 55 °C. The used antisense probes (vesicular glutamate transporter 1, *Vglut1 (produced 24.06.2015 primer for DNA inserts: 719 bp; Allen Brain Atlas Riboprobe ID: RP_050310_01_B09 (forward primer) FP:CAGAGCCGGAGGAGATGA; (reverse primer) RP: TTCCCTCAGAAACGCTGG, (nested 296 bp) FPnested: GCTGGCAGTGACGAAAGTGA, RPnested: TGAGAGGGAAAGAGGGCTGG)*, glutamate decarboxylase 1, *Gad1(67) (produced 24.06.2015 primer for DNA inserts: (320 bp) FP: GGCACGACTGTTTACGGAGC, RP: GCCTTGTCCCCGGTGTCATA))* were prepared in the hybridization buffer and heat-shocked at 95 °C for 2 min. Later, the probes were added to the sections and the hybridization took place at 55 °C overnight.

On the following days, the sections were washed in diluted SSC and a solution of diluted SSC and 50% formamide. Then, a solution of RNAse (4 µg/mL, Marcherey-Nagel, Düren, Germany) and 2 × diluted SSC was applied to get rid of residual RNA. Afterwards, the sections were once again washed with 2 × diluted SSC and a solution of SSC and 50% formamide. Afterwards, the sections were blocked in 4% normal goat serum in TBS (0.05 M, pH 7.5) at room temperature. After blocking, the sections were washed in TBS (0.05 M, pH 7.5) for 3 × 2 min at room temperature and 0.5% blocking solution (TSA Biotin System, Akoya Biosciences, Marlborough, Massachusetts, USA) was applied for 1 × 60 min at room temperature. The sections were then incubated in peroxidase-conjugated anti-digoxigenin (1:2000 in 0.5% blocking solution) at 4 °C overnight.

After the overnight incubation, the sections were washed in TBS (0.05 M, pH 7.5) for 3 × 10 min at room temperature, then biotinylated for 210 min at room temperature with biotin amide (200 µL/slice/well). After biotinylation, the sections were washed in TBS (0.05 M, pH 7.5) for 3 × 5 min at room temperature and streptavidin-conjugated AlexaFluor 488 1:400 (Life Technologies, Darmstadt, Germany) in TBS (0.05 M, pH 7.5) for 1 × 15 min at room temperature. Finally, the sections were washed in TBS (0.05 M, pH 7.5) for 3 × 5 min at room temperature.

Since the *FISH* protocol completely quenched the tdTomato fluorescence, an immunohistochemical staining was performed to recover the native signal. The sections were washed 3 × 15 min in TBS pH 7.5 and blocked for 90 min with 10% normal goat serum in TBS pH 7.5. The primary antibody rabbit-anti-RFP (Rockland, Philadelphia, Pennsylvania, USA) was diluted in the blocking solution to 1:500, incubated for 72 h at 4 °C. The sections were then washed 4 × 15 min with TBS pH 7.5 at room temperature. After washing, the sections were incubated in the secondary antibody, goat-anti-rabbit Alexa 594 (Life Technologies, Darmstadt, Germany) diluted 1:500 in TBS pH 7.5 for 4 h at room temperature. The sections were washed after this incubation for 3 × 10 and 3 × 20 min in TBS pH 7.5 at room temperature. To display the cell nuclei, sections were stained with 4’-5-diamidino-2-phenylindole (DAPI) diluted 1:1000 in TBS pH 7.5 for 5 min at room temperature. After staining the nuclei, the sections were washed 3 × 15 and 2 × 15 min in TBS pH 7.5. Then the sections were mounted on slides and covered with coverslips coated with Aqua Poly-Mount.

### Image acquisition and data analysis

Analysis and cell counting on brain sections was carried out with a fluorescence microscope (AxioImager.M2, Zeiss, Jena, Germany) with structured illumination (ApoTome, Zeiss). Cell counting was performed blinded. Further images were taken with the confocal microscope (Zeiss LSM 880) for illustration. For each probe or antibody, a 3D-images stack of the barrel cortex, identified by DAPI staining with a 25 × objective at an optical z-resolution of 1 µm were taken. In this 3D image, a 1000 µm-wide area through the barrel cortex was chosen for analysis and the cells were counted with Neurolucida (MBF Bioscience, Williston, Vermont, USA). Fluorescently-labeled cells of interest in this area were counted in a user-dependent manner if they were completely or (at the border) more than 50% within the delineated area. The DAPI staining enabled to delineate the cortical layers according to standard cytoarchitectonic criteria. The layer-specific analysis of the counted cells was carried out by Neuroexplorer (MBF Bioscience). The program reports the number of counted cells according to their defined markers and calculated the area of each layer and the total area in the chosen area of the barrel cortex in µm^2^. The calculated cell numbers were normalized in each section to 1 mm^3^ barrel cortex as described in^12^. In brief, first we multiplied the size of the total area (µm^2^) by the thickness of the Sect. (40 µm). This volume (in µm^3^) was scaled-up to 1 mm^3^ barrel cortex using a normalization factor, which was applied to the counted cell numbers in the total area, which made cell quantification from individual section comparable to each other. The same factor was also used to extrapolate the number of cells for each layer individually, so that the resulting values reflect the number of cells in their respective layer in a volume of 1 mm^3^ cortex (number of cell/mm^3^), to allow for a comparison across samples. A total of 26,014 cells were counted.

The layer-specific difference in colocalization between the two mouse lines was calculated with a Mann–Whitney-U non-parametric test with the statistics software GraphPad PRISM (GraphPad Software Inc, Boston, Massachusetts, USA). Descriptive statistics (mean, standard deviation, median, 25% percentile, 75% percentile, minimum, maximum) were calculated with the statistic software SigmaPlot (SYSTAT Software Inc, Palo Alto, California, USA).

### Supplementary Information

Supplementary Information.

## Data Availability

All data are available upon request from the corresponding author.
