# Supplementary material for: Intersectional strategy to study cortical inhibitory parvalbumin-expressing interneurons
Source: Sci Rep. 2024 Feb 3;14:2829. doi: 10.1038/s41598-024-52901-y (PMC10838283; doi:10.1038/s41598-024-52901-y)
Supplement: Supplementary file 1 — Supplementary Information. [file 41598_2024_52901_MOESM1_ESM.docx]

**
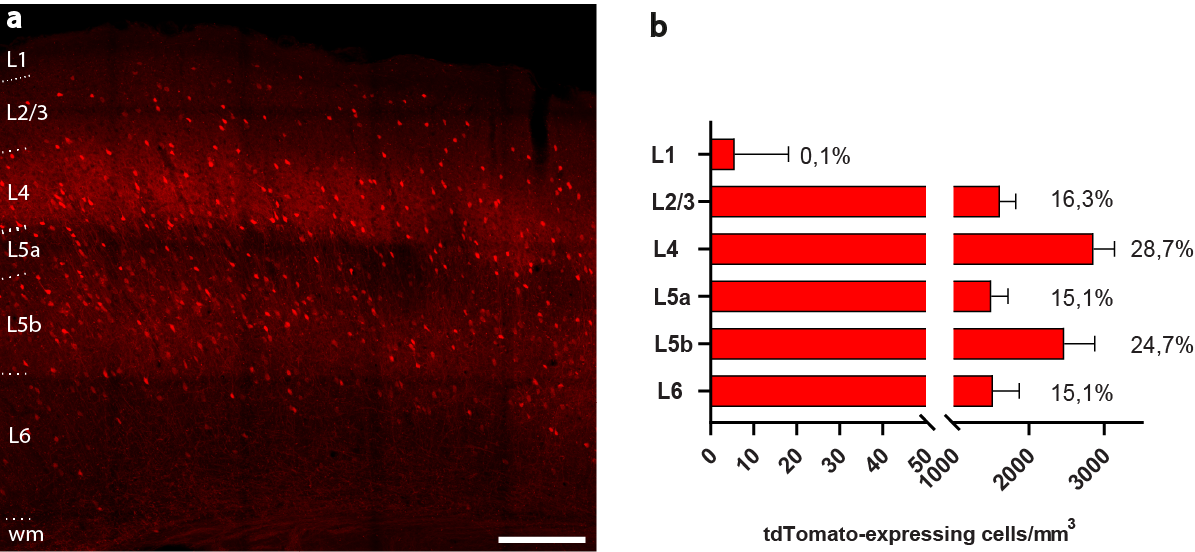
**

**Supplementary Fig. 1:** **Layer-specific distribution of parvalbumin-expressing cells in the barrel cortex of the *PV-Cre/tdTomato* mouse. a)** Overview image showing immunoamplified transgenic tdTomato. wm: white matter. Scale bar: 200 µm **b)** Layer-specific distribution of the tdTomato-expressing cells.

**
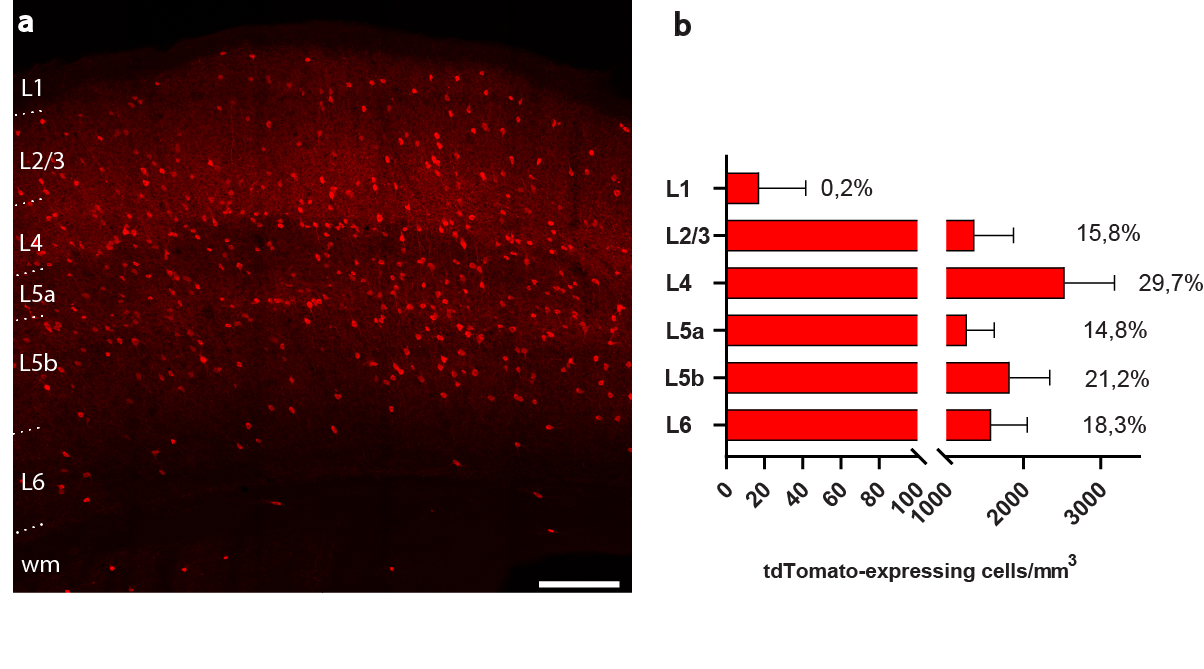
**

**Supplementary Fig. 2:** **Layer-specific distribution of parvalbumin-expressing cells in the barrel cortex of the *Vgat-Cre/PV-Flp/tdTomato* mouse. a)** Overview image showing immunoamplified transgenic tdTomato. wm: white matter. Scale bar: 200 µm **b)** Layer-specific distribution of the tdTomato-expressing cells.
